# A survey of general practitioners’ knowledge and clinical practice in relation to valvular heart disease

**DOI:** 10.1007/s11845-021-02619-x

**Published:** 2021-04-24

**Authors:** John P. Birrane, Zi Lun Lim, Chee H. Liew, Liesbeth Rosseel, Adrienne Heerey, Kieran Coleman, Joseph Gallagher, Darren Mylotte, John W. McEvoy

**Affiliations:** 1 University Hospital Galway and SAOLTA University Health Care Group; National University of Ireland, Galway, Ireland; 2 National Institute for Prevention and Cardiovascular Health, National University of Ireland Galway, Galway, Ireland; 3 Lower Salthill Medical Practice, Salthill, Galway, Ireland; 4 Irish College of General Practitioners, Lincoln Place, Dublin 2, Ireland

**Keywords:** General practice, Primary care, Specialist referral, Valvular heart disease

## Abstract

**Introduction:**

General practice has a key role in diagnosing patients with valvular heart disease (VHD) and referring them to appropriate services.

**Methods:**

An anonymous survey was conducted to assess the knowledge and clinical practice behaviour of Irish general practitioners (GPs) in relation to VHD. In addition to demographic data, the survey captured information in the following domains: knowledge of VHD prevalence, knowledge of contemporary VHD treatments, barriers to diagnosis, and referral patterns. To augment responses, a monetary prize (donated to charity) was offered and the survey was also disseminated using social media and by the Irish College of General Practitioners.

**Results:**

Valid survey responses were received from 197 GPs. The sample was well-balanced by gender, number of years in practice, and practice setting. A small proportion of GPs (16.8%) used a stethoscope to examine for VHD in all patients over 60 years, a figure that rose to 22.3% in patients over 75. Approximately half of participants (48%) felt confident in their ability to detect and diagnose VHD using a stethoscope, and 74% felt lack of access to echocardiography was a major barrier to making a VHD diagnosis. There was a high level of awareness among GPs of minimally invasive nonsurgical interventions now available for VHD treatment.

**Discussion:**

Irish GPs displayed good understanding of contemporary VHD treatment options but reported low confidence and inconsistent practices in evaluating patients for VHD. Improved access to echocardiography might help address these deficiencies, but reorganisation of services will be required in a resource-limited public health service.

**Graphical abstract:**

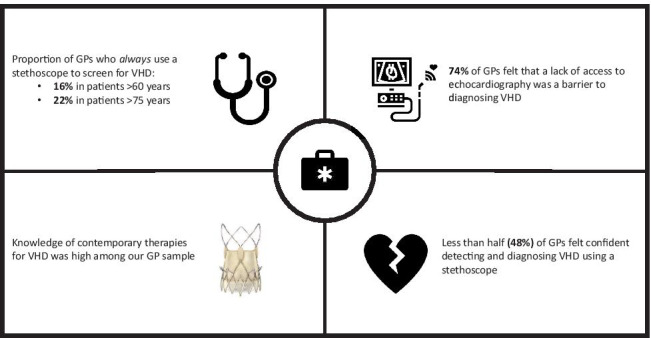

## Introduction

The prevalence of valvular heart disease (VHD) is increasing in tandem with an aging population and has been described by some commentators as ‘the next cardiac epidemic’ [1]. The predominant cause of VHD has shifted from rheumatic heart disease to degenerative valvular disease, the incidence of which increases with age [2]. Data from the USA indicates a prevalence of moderate to severe VHD in 8.5% of people aged between 65 and 74, rising to 13.3% in those aged 75 and over [3]. A UK community screening study of 2500 asymptomatic individuals > 65 years of age with no previous diagnosis of VHD detected some form of VHD in 50.8%, with 6.4% being diagnosed with VHD that was deemed clinically significant (moderate or severe) [4]. In another sample of 79,043 symptomatic UK adults referred for echocardiography, 14% had moderate to severe VHD [5]. This was remarkable given the authors sampled all adults aged 18 years or more. Such findings serve as evidence that VHD is increasingly a major public health problem.

Motivated by the pressing clinical need for VHD treatments among older and/or multimorbid patients, transcatheter-based interventions have recently been developed that provide minimally invasive therapeutic options for patients in whom surgical valve replacement (SVR) presents too high a risk. Compelling safety and efficacy data now exist for both transcatheter aortic valve implantation (TAVI) [6–10] in the treatment of aortic stenosis and transcatheter mitral valve repair in the treatment of mitral regurgitation [11]. These successes have spurned the development of promising transcatheter technologies designed to treat other VHD subtypes, like tricuspid regurgitation [12]. In the context of compelling evidence from such clinical trials, a number of tertiary cardiac centres in Ireland now perform transcatheter valve interventions, with outcomes that compare favourably to those observed in US and European registries [13–15].

Patient access to these interventions is dependent on appropriate referral. The European Heart Health Survey, carried out in 2015 and 2017, demonstrated a low level of knowledge about VHD among patients in Europe and low levels of physical examination for VHD by general practitioners (GPs), with both these findings being particularly evident in Ireland [16, 17]. Therefore, our objective was to survey a cohort of Irish GPs in more detail to obtain a better understanding of their knowledge and practice around VHD. There is also a need to understand the barriers that GPs face in diagnosing VHD, and investigate their patterns of referral to specialist care. By addressing these open questions, our survey may allow Irish health policy makers to better support GPs and hospital systems in caring for patients with VHD.

## Methods

This survey was developed and delivered by Croí and the National Institute for Prevention and Cardiovascular Health (NIPC). Both Croí and the NIPC are not-for-profit organisations based in the West of Ireland. This study was aimed at Irish GPs actively engaged in clinical practice anywhere in the Republic of Ireland. The survey was developed using an iterative process of review and refinement by content experts, representatives of the target population for the survey (GPs), and the primary investigators.

Survey questions were initially developed in consultation with a clinical cardiologist (JWMc) based in Galway University Hospital, a tertiary referral centre serving the west and north-west of Ireland. Questions were designed to assess the most clinically and scientifically important aspects of GP knowledge and practice in relation to VHD. Four GPs (two academic GPs and two community-based GPs) reviewed the questions for clarity and comprehensibility and submitted revisions that were incorporated into subsequent drafts of the survey. The study team then reviewed questions for brevity and concision, aiming for an overall completion time for the survey of ≤ 5 min. A draft survey was piloted at a GP conference on 8 February 2020, which further informed the final survey wording by integrating the feedback from attendees of this conference.

The survey consisted of 15 questions, 3 of which were demographic in nature: GP gender, years of clinical practice (< 10 years, 11–25 years, > 25 years), and practice setting (city, urban, rural, or mixed). The other survey questions are presented in Tables 1 and 2 and were designed to capture information within the following domains:

- Knowledge of VHD prevalence
- Knowledge of contemporary VHD treatments
- Barriers to diagnosis of VHD
- Referral patterns of patients with suspected VHD

**Table 1:** Survey items and responses

	Survey item	Responses	*n* (%)
1.1 Knowledge and practice related to valvular heart disease			
1	I feel confident that I can detect and diagnose moderate to severe valvular heart disease with my stethoscope (i.e., no echocardiography)	Strongly disagree	17 (8.6)
		Disagree	45 (22.8)
		Neither agree nor disagree	36 (18.3)
		Agree	91 (46.2)
		Strongly agree	8 (4.1)
2	Based on your clinical experience, is it worthwhile asking for salient symptoms in the for the detection of valvular heart disease?	No, the symptoms are too nonspecific and vague	24 (12.3)
		Yes, but taking a full history for all heart valve symptoms is only useful among patients who first complain of symptoms suggestive of heart valve disease	83 (42.6)
		Yes, all patients over 60 should be routinely screened for heart valve symptoms	88 (45.1)
3	In my clinical practice of patients > 60 years, I do a stethoscope examination to screen for valvular heart disease among asymptomatic patients	All the time > 50% of the time < 50% of the time Never	32 (16.2) 86 (43.7) 55 (27.9) 24 (12.2)
4	In my clinical practice of patients > 75 years, I do a stethoscope examination to screen for valvular heart disease among asymptomatic patients	All the time > 50% of the time < 50% of the time Never	44 (22.3) 85 (43.1) 48 (24.4) 20 (10.2)
5	Based on your clinical experience, what do you think the prevalence is of moderate or severe valvular heart disease in Irish adults between 60 and 74 years?	0–5% > 5–10% > 10–20% > 20%	63 (32) 70 (35.5) 54 (27.4) 10 (5.1)
6	Based on your clinical experience, what do you think the prevalence is of moderate or severe valvular heart disease in Irish adults ≥ 75 years	0–5% > 5–10% > 10–20% > 20%	12 (6.1) 67 (34) 69 (35) 49 (24.9)
1.2 Referral patterns and barriers to VHD diagnosis			
8	What do you see as being the barriers to diagnosing valvular heart disease?	Lack of direct access to echocardiography Waiting times for outpatient appointment Patient unable to attend outpatients	145 (74) 46 (23.5) 3 (1.5)
9	What percentage of patients with suspected asymptomatic valvular heart disease do you refer for a specialist opinion or echocardiography?	Less than 50% 50 to 75% More than 75% 100%	33 (16.8) 26 (13.3) 57 (29.1) 80 (40.8)
1.3 Knowledge regarding valvular intervention			
10	An elderly patient (> 75 years) with severe valvular heart disease must be able to undergo general anaesthesia and major surgery to be treated and those that cannot are not suitable for referral to a specialist	Strongly disagree Disagree Neither agree nor disagree Agree Strongly agree	99 (50.5) 67 (34.2) 16 (8.2) 8 (4.1) 6 (3.1)
11	The only treatment for severe symptomatic heart valve disease is surgical replacement of the valve	Agree Disagree Not sure	25 (12.8) 131 (66.8) 40 (20.4)
12	Frail patients and/or patients aged over 90 years are not candidates for heart valve replacement	Agree Disagree Not sure	45 (23) 98 (50) 53 (27)

**Table 2:** GP perceptions on the relative importance of screening for VHD and other cardiovascular disease (question 7 in the survey)

		Mean rank (*SD*)
Based on your clinical experience of patients over 60 years, please rank in order of importance from 1 (most important) to 5 (least important), the need to screen for the following medical conditions	Hypertension	1.71 (1.08)
	Atrial fibrillation	2.28 (.84)
	Diabetes	2.46 (.93)
	Heart valve disease	4.04 (.73)
	Abdominal aortic aneurysm	4.51 (.96)

Most survey items presented GPs with a set of responses from which they were to indicate the response that best fit their opinion. For some survey items, we utilised a Likert scale ranging from ‘strongly disagree’ to ‘strongly agree’. Other items asked GPs to choose a range of data that best fit their estimate regarding their own practice or a point of knowledge around VHD.

In preparing this manuscript, we complied with reporting guidelines from the equator-network (www.equator-network.org), focusing in particular on The Checklist for Reporting Results of Internet E-Surveys (CHERRIES) [18].

### Recruitment of participants

The survey was hosted on the SurveyMonkey platform. The NIPC has 175 GPs as ‘alliance members’ (an affiliate subscription membership that grants access to educational content produced by the NIPC) and these members were invited by email to complete the survey. A total of 152 GPs who had attended an NIPC webinar in July 2020 were also invited to complete the survey. In addition, the survey was disseminated by the Irish College of General Practitioners (the professional and training body for GPs) by inclusion within an ebulletin to their members. Furthermore, the Health Service Executive (HSE; the organisational body in charge of running the public health service in Ireland) emailed a link for the survey to GPs providing general medical services to public patients. Finally, the survey was circulated on social media (WhatsApp, Twitter, LinkedIn). GPs were encouraged to participate by offering entry into a draw for a donation of five hundred euros to be made to a charity of their choice.

### Statistical analysis

All survey data were exported from the SurveyMonkey platform to Microsoft Excel. Further statistical analysis was carried out using SPSS Version 26 (IBM, Armonk, New York). Categorical responses were reported as numbers and proportions. Continuous/numerical responses we reported as means and standard deviations. When comparing categorical responses between the various demographic groups, we used chi-squared testing. We considered a two-sided *p* < 0.05 as statistically significant.

## Results

In total, 199 survey responses were obtained. One participant’s data were excluded as he/she only responded to 1 of the 15 survey questions. Two consecutive responses on the same date were noted to have identical answers to each of the survey questions, raising suspicion of a duplicate. Data from one of these responses were excluded leaving 197 unique respondents for analysis. Owing to the use of varying approaches in disseminating the invitation for this survey (described in the ‘Methods’ section), inclusive of social media advertisements, is not possible to know exactly how many GPs reviewed the invitation to participate in our survey. Therefore, we are unable to accurately report a survey response rate.

Responses for each survey item are presented in Tables 1 and 2. Demographic characteristics of the survey participants are described in Table 3, demonstrating that the study sample was balanced in terms of sex, years of experience, and practice setting.

**Table 3:** Demographic data of survey participants (*N* = 197 General Practitioners)

	*n* (%)
Years’ experience	
< 10 years	61 (31)
11–25 years	63 (32)
> 25 years	73 (37)
Gender	
Men	94 (48)
Women	102 (52)
GP practice	
City	51 (26)
Urban	35 (17.9)
Rural	50 (25.5)
Mixed	60 (30.6)

### GP knowledge and practice

**Fig. 1:**
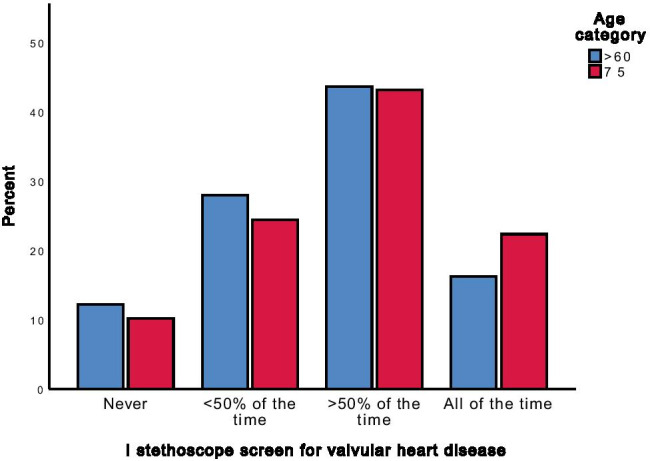
Frequency of stethoscope screening by GPs, by age category

Survey responses indicated that 16.2% of GPs always examined patients older than 60 for VHD with a stethoscope, a figure which increased to 22.3% in patients older than 75 years (Fig. 1). The proportion of GPs who never examined for VHD with a stethoscope was 12.2% for patients > 60 years and 10.2% for patients > 75 years. The proportion of GPs who examined patients with a stethoscope > 50% and < 50% of the time was similar whether they were > 60 years or > 75 years.

**Fig. 2:**
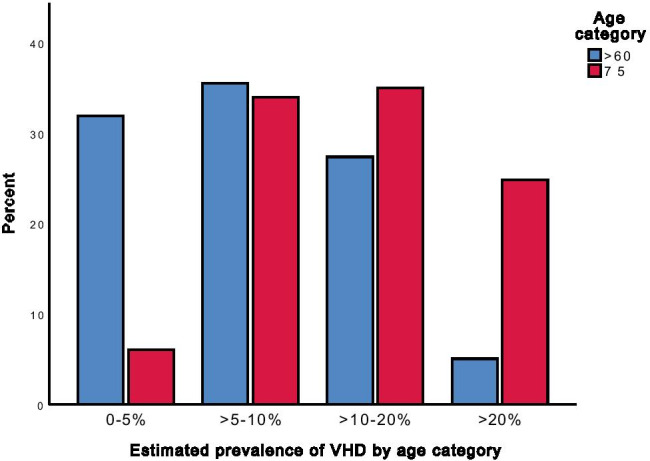
Estimated prevalence of VHD by age category

Most GPs correctly identified that the prevalence of VHD increases with age (Fig. 2), though the actual prevalence estimates varied widely among respondents and often differed from the correct values reported in the literature (which document a prevalence of moderate-severe VHD of 5–10% among adults 60–74 years old and 10–20% among adults aged 75 or older).

**Fig. 3:**
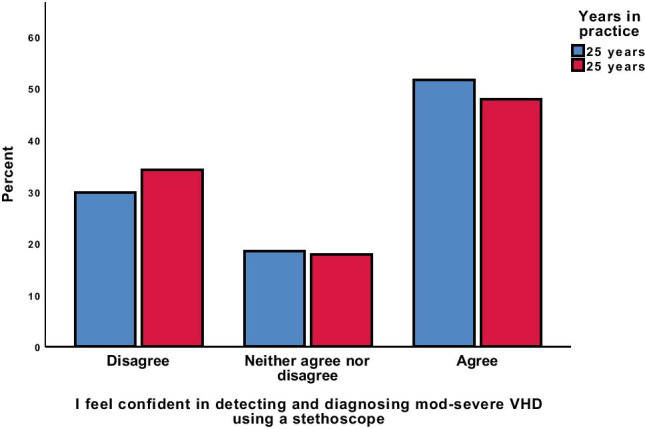
Confidence in diagnosing VHD with a stethoscope divided by years in practice

Approximately half of GPs surveyed (50.3%) reported confidence in detecting and diagnosing moderate-severe VHD by auscultation of the praecordium with a stethoscope. By contrast, 31.4% reported that they were not confident in diagnosing VHD with a stethoscope, while 18.3% of doctors were uncertain in their diagnostic ability. We found no difference in reported confidence in detecting and diagnosing VHD with stethoscope examination in GPs that had been practicing for ≤ 25 years or > 25 years (Fig. 3). The vast majority (87.7%) of GPs agreed that asking for salient symptoms was important for the detection of VHD, but only 45% reported doing so routinely in patients ≥ 60 years old.

We also asked GPs to rank the importance of screening for VHD relative to four other common cardiovascular conditions (Table 2). GPs ranked hypertension, atrial fibrillation, and diabetes, in that order, ahead of valvular heart disease in relative importance for screening. Valvular heart disease (*M* = 4.04, *SD* = 0.73) was ranked slightly ahead of abdominal aortic aneurysms (*M* = 4.51, *SD* = 0.96) as a priority for screening.

### Referral patterns and barriers to diagnosis of VHD

Three quarters of Irish GPs reported that a lack of access to echocardiography was a barrier to diagnosing VHD in their patients. Moreover, 23.5% indicated that waiting times for specialist outpatient appointments were also considerable barriers to VHD detection in their practice. Despite this, almost 70% of GPs nevertheless reported sending the majority of asymptomatic patients that they suspected of VHD for echocardiography or a specialist opinion.

### Knowledge regarding VHD intervention

Most GPs felt that a patient with severe VHD unable to tolerate general anaesthesia or open-heart surgery could still benefit from a specialist opinion. Two-thirds (66.8%) of GPs disagreed that SVR was the only intervention for severe symptomatic VHD, indicating a familiarity with less invasive approaches. Just 25 (12.8%) of GPs believed that SVR was the only intervention for their patients with VHD. Similarly, 50% disagreed with a statement that frail patients or those older than 90 years were not candidates for valve replacement compared with 23% who agreed (thereby indicating that half were aware of less invasive valve replacement options for the frail and very old).

**Fig. 4:**
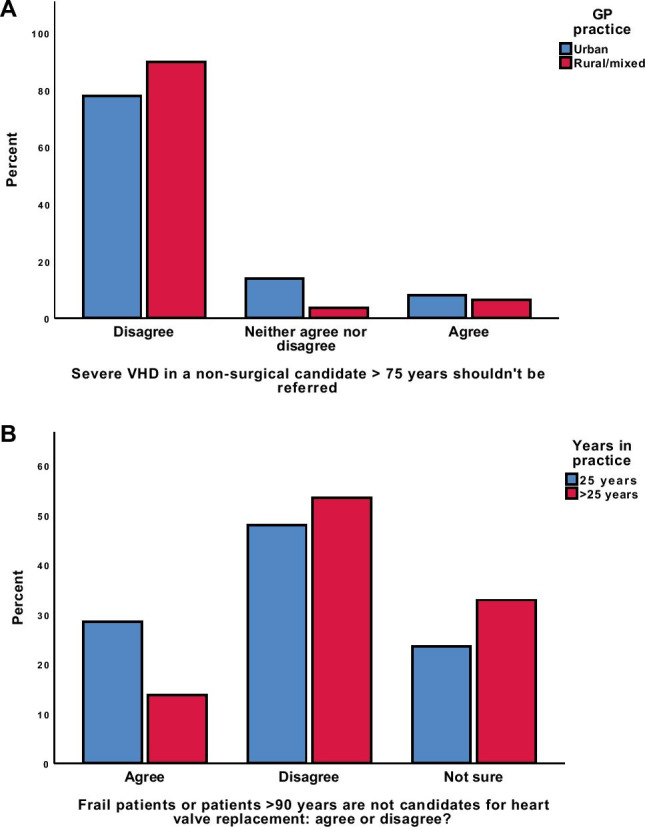
Differences in knowledge of intervention in VHD on the basis of selected demographic subgroups. **a** Responses based on location of GP practice. **b** Responses based on years in practicecxs

When survey responses were subdivided by demographic factors (Table 4; Fig. 4), a slightly higher number of GPs from rural and mixed practices indicated an awareness that older and frail patients are eligible for valve replacements, that there were less-invasive alternatives to SVR, and that severe symptomatic VHD patients who were not surgical candidates should still be referred for specialist opinion. A chi-squared analysis of these responses indicated a significant difference (*p* = 0.03) in the responses of GPs to question number 10 (see Table 1), where GPs were asked if they agreed there were alternatives to SVR in a frail, multimorbid patient. However, inspection of the standardised residuals revealed that this difference lay predominantly in the proportion of GPs who were unsure.

**Table 4:** Knowledge regarding valvular intervention, divided by urban and rural practitioners, and by years of experience

		Urban/mixed *n* (%)	Rural *n* (%)	≤ 25 years *n* (%)	> 25 years *n* (%)
An elderly patient (> 75 years) with severe valvular heart disease must be able to undergo general anaesthesia and major surgery to be treated and those that cannot are not suitable for referral to a specialist	Agree	7 (8.1)	7 (6.4)	8 (6.5)	6 (8.2)
	Not sure	12 (14)	4 (3.7)	13 (10.6)	3 (4.1)
	Disagree	67 (77.9)	98 (89.9)	102 (82.9)	64 (87.7)
		$\chi$^2^ (2) = 7.21, *p* = 0.03		$\chi$^2^ (2) = 2.65, *p* = 0.27	
The only treatment for severe symptomatic heart valve disease is surgical replacement of the valve. Please choose from the options below:	Agree	12 (14)	13 (11.9)	11 (8.9)	14 (19.2)
	Not sure	17 (19.8)	22 (20.2)	25 (20.3)	15 (20.5)
	Disagree	57 (66.3)	74 (67.9)	87 (70.7)	44 (60.3)
		$\chi$^2^ (2) = .18, *p* = .92		$\chi$^2^ (2) = 4.51, *p* = .11	
Frail patients and/or patients aged over 90 years are not candidates for heart valve replacement. Please choose one of the options below:	Agree	22 (25.6)	23 (21.1)	35 (28.5)	10 (13.7)
	Not sure	22 (25.6)	31 (28.4)	29 (23.6)	24 (32.9)
	Disagree	42 (48.8)	55 (50.5)	59 (48)	39 (53.4)
		$\chi$^2^ (2) = .59, *p* = 0.75		$\chi$^2^ (2) = 6.08, *p* = 0.05

We also investigated whether years of experience was associated with GPs’ awareness of newer, less invasive interventions, hypothesising that more recently trained GPs may have encountered transcatheter valve interventions during hospital rotations, prior to entering full-time GP practice. For the purpose of this analysis, we divided GPs according to whether they had greater than or equal to 25 years of experience, and those who had less than 25 years’ experience. A higher proportion of the more experienced GP group (53.4%) correctly noted that frail or older patients may be candidates for valve replacement in the current era of less invasive therapeutic interventions, compared with GPs in the lower experience group (48%), a finding that was statistically significant ($\chi$^2^ (2) = 6.08, *p* = 0.048) (Table 4).

## Discussion

We note the following observations from our survey of Irish GPs: (1) Only 50% of GPs were confident they could detect and diagnose VHD using a stethoscope. (2) Only 45% of GPs agree that symptoms of VHD should be routinely queried among patients > 60 years old. (3) GPs correctly recognised that VHD prevalence increases with age, but many under- or over-estimated of the true prevalence of VHD, the best estimate for which is 5–10% in patients 65–74 years old and 10–20% in those older than 75 [3, 4]. (4) While less than a quarter of GPs reported routine use of a stethoscope exam to examine for VHD among patients ≥ 60 years old, GPs were more likely to examine all patients ≥ 75 years of age. And (5) GPs appeared to demonstrate excellent knowledge of newer therapeutic options for VHD and recognised that frail and older patients should still be referred for specialist opinion given the contemporary availability of less invasive therapeutic interventions.

Our survey also demonstrates that there is concordance between GPs’ self-reported practice with respect to VHD and the previously surveyed experience of Irish patients. The previously published finding that 68% of Irish patients over 60 years infrequently had their chest examined with a stethoscope [16, 17] is broadly consistent with our finding that 27.9% of GPs reported examining patients ≥ 60 years with a stethoscope exam less than half of the time and that 12.2% of them reported never doing so. Furthermore, our survey indicates that this may be related to the low levels of self-reported confidence among GPs in their ability to detect and diagnose VHD by physical examination.

Previous research into the accuracy of physical examination in detecting and diagnosing VHD have reported conflicting results owing to heterogeneity of the doctor and patient characteristics of the cohorts studied. A 2016 review presented supporting evidence for the value of physical examination in the diagnosis of VHD, but many of the cited studies featured cardiologists as the examining doctors [19]. By contrast, a 2018 study of GPs found that the sensitivity of cardiac auscultation for diagnosing VHD in asymptomatic patients was poor (44% for clinically significant VHD) when conducted within a large, community-based screening programme [20].

While physical examination is an accessible and inexpensive method for the detection of murmurs resulting from VHD, in addition to facilitating the bond between doctor and patient [21], echocardiography is the gold standard for the diagnosis of VHD and for the quantification of the severity of the condition. About 74% of GPs in this survey reported a lack of direct access to echocardiography as being a barrier to diagnosing VHD. Initiatives to increase the availability of echocardiography in the community are in the process of being implemented in Ireland as part of the Slaintecare programme [22]. Judging by the results of this study, such initiatives will be welcomed by GPs. We look forward, in time, to research detailing their effects on community-based diagnosis and management of VHD.

Ultrasound and echocardiography are increasingly a feature of modern medical school curricula [23]. A number of studies have demonstrated satisfactory acquisition of ultrasound skills among medical students and enhancement of diagnostic accuracy after training with the technology [24–28]. Opportunistic screening (i.e., case-finding) using focused point-of-care ultrasound by GPs may represent a means of enhancing detection rates in the primary care environment. Full departmental echocardiograms could then be reserved for cases with higher indexes of suspicion or provisional abnormalities detected by GPs at the point-of-care. Such an approach could plausibly reduce waiting times for outpatient echocardiography and ensure that urgent cases were detected in good time, particularly given GPs’ low confidence in diagnosing VHD using stethoscope alone. Based in part on our survey results, professional bodies responsible for the continuing professional development of GPs might consider applying established medical school curricula to educational offerings designed for the up-skilling of interested GPs in point-of-care ultrasound of the heart. Whether such efforts are practical requires investigation. Alternatively, new artificial intelligence techniques offer the potential to augment the GP’s ability to diagnose VHD using the stethoscope [29, 30].

Awareness among GPs regarding alternatives to surgical replacement of diseased valves was generally high in this survey. This is an important and encouraging result from our survey because new transcatheter valvular interventions have removed a significant barrier to effective treatment among patients who cannot tolerate general anaesthesia or open heart surgery. Nonetheless, further education of GPs regarding these new technologies is motivated by the finding that 12.8% of the sample appeared to not recognise that there were alternatives to traditional open-heart surgery. A significant minority of GPs (23%) also did not agree that frail patients and/or patients aged over 90 years were candidates for heart valve replacement. Interestingly, GPs from rural and mixed settings appeared more likely to indicate an awareness that less invasive therapeutic interventions are now available to older, frail patients. This might reflect a greater familiarity with the older patient cohorts generally found in rural settings [31], and experience of their patients having had successful intervention.

Limitations of the present study include the fact that the sample of GPs was self-selected. Owing to the methods of dissemination deployed, which included web-based dispersal of a web link to the survey, we were unable to calculate an accurate response rate for the study. This is because the denominator number of how many GPs actually read the invitation (whether or not they participated) was impossible to determine. On the basis of the demographic characteristics observed; however, we had a good representation of GPs across age, gender, and practice setting. Another limitation is that a small proportion of GPs who responded completed the survey after a webinar that included a talk on VHD. Therefore, knowledge levels may have been higher in our respondents compared to a sample of GPs who have not recently undertaken a continuing professional development in cardiology. Finally, it is possible in retrospect that the wording for question number 1 caused confusion among some GPs in that they may have taken the question to relate to ‘any’ form of moderate to severe VHD or they may have interpreted the question to ask about their confidence in diagnosing a specific subtype of VHD (e.g. mitral stenosis vs mitral regurgitation vs other).

In conclusion, we observed low confidence and inconsistent practices in the evaluation of VHD in this sample of Irish GPs. On a more positive note, respondents demonstrated an awareness regarding the association of increasing age with VHD and good knowledge regarding contemporary treatment options. Improved access to echocardiography, either at the point of care in the form of limited ultrasound examinations or in the hospital/cardiology clinic in the form of full examinations, along with the development of a structured approach to evaluating patients for VHD has the potential to enhance its detection and diagnosis in the community.
